# Multivariable analysis of clinical and laboratory data manifestations predicting severity and mortality risk in patients with Coronavirus disease 2019 in the mountainous west of Iran: a retrospective single-center study

**DOI:** 10.2478/abm-2022-0005

**Published:** 2022-02-28

**Authors:** Zahra Naderi Beni, Afsaneh Naderi Beni, Fereidoun Rahmani Samani, Mohammad Ali Dayani, Fariba Naderi Beni, Hamed Radmehr, Pegah Noorshargh

**Affiliations:** Shahrekord University of Medical Sciences, Shahr-e Kord, Chaharmahal and Bakhtiari Province 88167-54633, Iran; Isfahan Eye Research Center, Isfahan University of Medical Sciences, Isfahan 81496-44874, Iran

**Keywords:** COVID-19, clinical decision-making, clinical laboratory techniques, Iran, pneumonia

## Abstract

**Background:**

Few reports have addressed the clinical and laboratory features of patients with coronavirus disease-2019 (COVID-19) in mountainous areas, especially in Iran.

**Objectives:**

To report the clinical and laboratory data and manifestations predicting mortality of patients with COVID-19 in the west of Iran.

**Methods:**

We conducted a retrospective cohort study of 286 patients hospitalized with COVID-19 between 25 February 2020 and 12 May 2020 to describe their clinical symptoms and laboratory test findings when they were admitted at the Hajar Hospital affiliated with the Shahrekord University of Medical Sciences, and a multivariable analysis of factors that predict their disease severity and mortality.

**Results:**

After hospital admission, 18 patients died and 268 were discharged. Older age [odds ratio (OR) = 1.02, 95% confidence interval (CI) = 1.01–1.04, *P* = 0.001], presence of underlying diseases (OR = 1.86, 95% CI = 1.01–3.45, *P* = 0.04), elevated hematocrit (OR = 1.08, 95% CI = 1.03–1.13, *P* = 0.002), and increase in red blood cell distribution width (RDW) coefficient of variation (OR = 1.18, 95% CI = 1.02–1.36, *P* = 0.02) were significantly associated with disease severity. Older age (OR = 1.00, 95% CI = 1.00–1.07, *P* = 0.03), hypocalcemia (OR = 0.20, 95% CI = 0.09–0.58, *P* = 0.002), hypophosphatemia (OR = 0.50, 95% CI = 0.26–1.02, *P* = 0.04), and increase in platelet-larger cell ratio (P-LCR; OR = 1.10, 95% CI = 1.00–1.15, *P* = 0.04) were significantly associated with mortality. The areas under the receiver operating characteristic curves were as follows: calcium 0.759; lactate dehydrogenase (LDH) 0.731; phosphorus 0.725; bilirubin 0.689; C-reactive protein 0.679; and RDW – standard deviation (RDW-SD) 0.624.

**Conclusions:**

Those who did not survive tended to be elderly and had a greater incidence of comorbidities. Elevated LDH, decreased levels of calcium and phosphorus, and anemia at diagnosis were associated with greater risk of death for these Iranian patients hospitalized with COVID-19. Regular assessment of these markers would help to manage patients with COVID-19.

At the end of 2019, pneumonia due to the severe acute respiratory syndrome coronavirus 2 (SARS-CoV-2, formerly known as 2019-nCoV) emerged in China and rapidly spread to other countries [1, 2]. The World Health Organization (WHO) declared the coronavirus disease-2019 (COVID-19) as a global pandemic on March 11, 2020 [3]. The disease is highly transmissible and has spread rapidly worldwide [1, 2]. Situated at an altitude of 2,050–2,310 m in the western region of Iran, the provincial capital Shahr-e Kord was one of the last cities with confirmed cases of COVID-19.

Although considerable time has passed since the emergence of COVID-19, the clinical features and laboratory characteristics of COVID-19 are not yet fully clear [4]. Few reports have addressed the clinical and laboratory features of patients with COVID-19 in mountainous areas, especially in Iran [5,6,7,8,9,10,11]. The present study aimed to analyze the clinical signs and symptoms, as well as clinical laboratory parameters, to determine the risk factors for outcomes of hospitalized patients confirmed with COVID-19 in Shahr-e Kord, Chaharmahal and Bakhtiari Province, Iran.

## Methods

### Study design

Ethical approval for the present study was obtained from the institutional review board of the Shahrekord University of Medical Sciences, and the protocols used in this study were approved by the Research Ethics Committee (approval ID No. IR.SKUMS.REC.1399.008). The study was conducted according to the ethical principles of the contemporary revision of the Declaration of Helsinki (64th WHA General Assembly, 2013) and the national norms and standards for conducting medical research in Iran. We conducted a retrospective study of a cohort of all patients hospitalized with COVID-19 between February 25 and May 12, 2020. Informed consent was obtained from the study participants or the nearest family members of children aged <18 years or deceased patients. Reporting is in accordance with the guidelines of the Strengthening the Reporting of Observational Studies in Epidemiology (STROBE) statement [12].

### Study setting

The present study was conducted at the Hajar Hospital in Shahr-e Kord, which is a major referral and teaching hospital of the Shahrekord University of Medical Sciences, and is a designated COVID-19 center in Chaharmahal and Bakhtiari Province, Iran.

### Study population

All patients with clinically or laboratory-confirmed [reverse transcription polymerase chain reaction (RT-PCR)-positive] cases of COVID-19 [3], who were discharged or who died at Hajar Hospital between February 25 and May 12, 2020, were included in this study.

### Variable definitions

Fever was defined as having an axillary temperature of 37.5 °C or higher. Laboratory testing, including a complete blood count, blood chemistry tests, coagulation tests, evaluation of liver and kidney functions, and measurement of serum levels of electrolytes, C-reactive protein (CRP), lactate dehydrogenase (LDH), and creatine kinase. Lymphocytopenia is defined as a lymphocyte count <1,500 cells per mm^3^. Thrombocytopenia is defined as platelet (PLT) count <150,000 cells per mm^3^. In all patients with COVID-19 who recovered, clinical signs and symptoms resolved fully and oxygen saturation levels improved substantially. These patients had not developed extrapulmonary organ dysfunction and did not require longer supportive care.

RT-PCR assays were performed using a Novel Coronavirus (2019-nCoV or SARS-CoV-2) RT Multiplex RT-PCR Kit (Liferiver; Shanghai ZJ Bio-Tech Co.) according to the manufacturer's instructions and the WHO protocol [13].

High-resolution computed tomography (HR-CT) findings were categorized as normal and abnormal. Intubation and mechanical ventilation needing was classified as yes or no. We considered the patients’ outcomes as discharged and died.

### Data sources

We collected data from the medical records and compiled data from patients hospitalized with COVID-19, confirmed by a positive result by RT-PCR assay of a nasopharyngeal swab specimen or clinical diagnosis criteria based on typical CT imaging findings.

Clinical, laboratory, and outcome data were extracted from electronic medical records. Chest X-ray imaging and thoracic CT and all clinical laboratory testing were performed according to the patient's clinical care needs. The medical record data consist of patients’ basic information, signs and symptoms, laboratory test results, other comorbidities, medications prescribed, and outcomes.

Based on medical record documentation or description, and sometimes, review of imaging scans, radiographic abnormality was determined.

All registered cases at the hospital had a unique national code, so there were no duplicates.

### Statistical analysis

Data are described using mean and standard deviation (SD) for normally distributed continuous variables, the median and interquartile range (IQR) for non-normal variables, and number and percentages for categorical variables. We assessed differences between survivors and those who did not survive using a Student *t* test for continuous data, a χ^2^ test for categorical data, and nonparametric methods for highly skewed distributions. An additional analysis of the moderate and severe subgroups (severity according to the Seventh Edition of the *Diagnosis and Treatment Protocol for COVID-19 Patients*, the National Health Commission of China) was conducted [14].

Univariable and multivariable regression analyses were used to describe the association between demographic and laboratory data and in-hospital death and disease severity. Receiver operating characteristic (ROC) curves depicted the accuracy of the laboratory parameters in predicting the prognosis of patients with COVID-19 and were evaluated by calculating the area under the curves (AUCs). *P* < 0.05 was considered significant. The statistical analyses were performed using Stata 9.1 statistical software (Stata Corp.).

## Results

**Figure 1:**
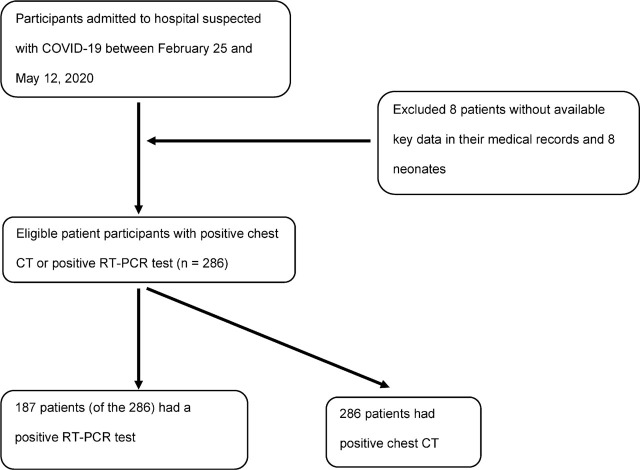
Flowchart for the inclusion and exclusion criteria for the patient samples. COVID-19, coronavirus disease-2019; CT, computed tomography; RT-PCR, reverse transcriptase-polymerase chain reaction.

At Hajar Hospital, Shahr-e Kord, Iran, 302 patients were hospitalized with COVID-19 before 12 May 2020. We excluded data from 8 patients with incomplete available data in their medical records and from 8 neonates. Finally, we included data from 286 inpatients (**Figure 1**). During their hospital stay, 18 patients died, and 268 were discharged from hospital. Their demographic characteristics and clinical features are shown in **Table 1**. The median age of the patients was 52 years (IQR, 36–71 years); 6.2% of the patients were <15 years old. A total of 44.7% were female. Comorbidities were found in 47.2% of the patients and included hypertension (28.0%) as the most prevalent, followed by ischemic heart disease (17.8%), diabetes mellitus (15.0%), neurological disease (5.6%), pneumonia, asthma, kidney disease (i.e., urine albumin-to-creatinine ratio (UACR) ≥30 mg/g and/or an estimated glomerular filtration rate [GFR] <60 mL/min/1.73 m^2^), and thyroid disorders.

**Table 1:** Demographic data and baseline characteristics at admission of patients infected with COVID-19 who survived and those who did not

**Variable** n (%) or median (IQR)	**All patients** **n = 286**	**Moderate** **n = 134**	**Severe** **n = 152**	***P***	**Survived** **n = 268**	**Did not survive** **n = 18**	***P***
Age (range) years	53 (36, 71)	43 (29, 67)	61 (46, 74)	<0.001	52 (35, 70)	73 (60, 79)	0.001
0–14 years	18	15	3		18	0	
15–49 years	105	63	42		105	0	
50–64 years	56	26	30		51	5	
≥65 years	107	30	77		94	13	
Female/male	128/158	61/73	67/85	0.94	120/148	8/10	0.37
Fever on admission	155 (54.1)	75 (56)	80 (53)	0.59	145 (54)	10 (56)	0.85
Myalgia or arthralgia	76 (26.5)	36 (27)	40 (26)	0.89	70 (26)	6 (33)	0.60
Cough	177 (61.9)	87 (65)	90 (59)	0.53	154 (57)	14 (78)	0.21
Dyspnea	144 (50.3)	70 (52)	74 (48)	0.41	133 (50)	11 (61)	0.45
Confusion	31 (10.8)	17 (13)	14 (9)	0.33	25 (9)	6 (33)	0.09
Seizure	1 (0.4)	1 (1)	0 (0)	0.28	0	1 (6)	0.69
Headache	134 (46.8)	69 (52)	67 (44)	0.35	121 (45)	13 (72)	0.27
Chest pain	41 (14.3)	19 (14)	22 (15)	0.96	36 (13)	5 (28)	0.27
Abdominal pain	19 (6.6)	11 (8)	8 (5)	0.31	18 (7)	1 (6)	0.28
Nausea	32 (11.1)	14 (10)	18 (12)	0.72	31 (12)	1 (6)	0.19
Vomiting	23 (8.04)	13 (10)	10 (7)	0.32	22 (8)	1 (6)	0.20
Diarrhea	5 (1.7)	3 (2)	2 (1)	0.64	4 (2)	1 (6)	0.20
Comorbidity	135 (47.2)	48 (36)	87 (57)	0.006	121 (45)	14 (78)	0.007
Cancer	5 (1.7)	2)2)	3 (2)	0.76	4 (2)	1 (6)	0.25
Liver	2 (0.7)	2 (2)	0 (0)	0.06	2 (1)	0	0.69
Diabetes mellitus	43 (15.0)	12 (9)	31 (20)	0.02	38 (14)	5/15 (28)	0.19
Hematology	4 (1.4)	2 (2)	2 (1)	0.48	4 (2)	0	0.58
Pregnancy	7 (2.4)	7 (5)	0 (0)	0.007	7/259 (3)	0	0.026
Heart disease	51 (17.8)	17 (13)	34 (22)	0.15	45 (17)	6 (33)	0.14
Hypertension	80 (28.0)	17 (13)	63 (42)	0.014	75 (28)	5 (28)	0.86
Asthma	9 (3.1)	5 (4)	4 (3)	0.058	9/257 (3)	0	0.40
COPD	38 (13.3)	9 (7)	29 (19)	0.006	35 (13)	3 (17)	0.80
Neurology	16 (5.6)	7 (5)	9 (6)	0.8	14 (5)	2 (11)	0.37
Other	40 (14.0)	15 (11)	25 (17)	0.26	39 (15)	1 (6)	0.23
Time from illness onset to first admission	4 (3, 7)	4 (2, 6)	4 (3, 7)	0.023	4 (3, 6)	7 (3, 10.5)	0.045
Time from admission to discharge/death	15 (14, 19)	15 (13, 18)	15 (14, 19)	0.047	15 (14, 18)	18 (14, 21.5)	0.031
First oxygen mode							
No oxygen inhalation	144 (50.3)	91 (68)	0 (0)	<0.001	143 (53)	1 (10)	<0.001
Nasal catheter for oxygen	117 (40.9)	43 (32)	130 (86)		113 (42)	4 (22)	
Face mask oxygen inhalation	16 (5.6)	0	17 (11)		12 (4)	4 (22)	
High-flow oxygen	5 (1.7)	0	5 (3)		0	5 (28)	
Noninvasive ventilation	4 (1.4)	0			0	4 (22)	
Tracheal intubation	0	0			0	0	

COPD, chronic obstructive pulmonary disease; COVID-19, coronavirus disease-2019; IQR, interquartile range.

The mean interval between hospital admission to discharge was 16.4 [95% confidence interval (CI) 16, 16.9] d (median: 15, IQR: 14, 19), ranging from 5 d to 40 d. Cough was the most frequent observed symptom (61.9%), followed by fever (54.1%), myalgia (26.5%), nausea and vomiting (19.2%), and headache (11.9%). Of the 20 patients who required mechanical ventilation, 18 (90%) patients died.

The presence of underlying comorbidities was more common in patients severely or critically ill with COVID-19 than in those patients with nonsevere disease (57% vs. 36%).

### Clinical outcomes

None of the 286 patients whose data were included in the study were lost to follow-up. At the end of the study primary composite end point event occurred in 33 patients (11.5%), including 11.5% who were admitted to the intensive care unit (ICU), 8.7% who required invasive mechanical ventilation, and 6.2% who died.

On admission, laboratory findings included lymphocytopenia, which was found in 55.2% of the patients, thrombocytopenia in 30.6%, and leukopenia in 15.6%. Erythrocyte sedimentation rate (ESR) was elevated in 60.6% of the patients. CRP levels were elevated in 35.7% of patients, and 69.8% had elevated alkaline phosphatase (ALK-p). Levels of aspartate aminotransferase (AST) were elevated in 31.1% of patients, and levels of alanine aminotransferase (ALT) were elevated in 18.2% of patients. We found no significant differences between survivors and deceased in terms of sex and lymphocyte count; however, significant differences were found for age, serum levels of calcium, LDH, and phosphorus, and red blood cell (RBC) counts on admission (*P* < 0.05) (**Table 2**).

**Table 2:** Laboratory findings at admission of patients infected with SARS-CoV-2

**Variable, median (IQR)**	**Total**	**Disease severity**			**Mortality**		
		
**Admission data**		**Moderate**	**Severe**	***P***	**Survived**	**Did not survive**	***P***
Albumin (g/dL)	3.2 (2.9, 3.5)	3.2 (2.83, 3.5)	3.2 (2.8, 3.5)	0.57	3.2 (2.9, 3.5)	3.0 (2.75, 3.5)	0.38
ALK-p (IU/L)	180 (142, 254)	172 (144, 276)	188 (138, 246)	0.90	180 (139, 262)	193 (144, 239)	0.90
Direct bilirubin (mg/dL)	0.2 (0.1, 0.4)	0.2 (0.1, 0.4)	0.2 (0.1, 0.4)	0.78	0.2 (0.1, 0.4)	0.4 (0.2, 0.6)	0.01^*^
Total bilirubin (mg/dL)	0.6 (0.4, 1.0)	0.5 (0.4, 0.82)	0.6 (0.4, 1.12)	0.29	0.5 (0.4, 0.9)	0.9 (0.65, 1.4)	0.01^*^
Calcium (mg/dL)	8.3 (8.0, 8.7)	8.3 (8.0, 8.7)	8.3 (8.0, 8.7)	0.56	8.4 (8.0, 8.7)	8.0 (7.2, 8.2)	<0.001^***^
Corrected calcium (mg/dL)	8.96 (8.56, 9.28)	8.94 (8.44, 9.26)	8.94 (8.57, 9.35)	0.56	8.98 (8.62, 9.34)	8.68 (7.91, 9.02)	0.01^*^
CRP (mg/L)	10.0 (4.0, 15.5)	8.0 (4.0, 16.0)	10.0 (3.5, 15.0)	0.90	9.5 (4.0, 16.0)	10 (2.0, 13.0)	0.68
ESR (mm/h)	30 (13, 51)	26 (13, 50)	33 (14.5, 53)	0.30	29 (13, 51)	32 (18.8, 53.3)	0.64
Hematocrit (%)	40.0 (36.1, 43.6)	39.8 (36.1, 42.8)	41.6 (36.4, 44.6)	0.027^*^	40.0 (36.1, 43.3)	41.1 (36.0, 44.9)	0.53
Hemoglobin (g/dL)	13.8 (11.8, 15.2)	13.4 (12.0, 14.9)	14.0 (11.9, 15.4)	0.093	13.8 (11.9, 15.1)	14.5 (11.4, 16.3)	0.54
LDH (U/L)	568 (411, 839)	466 (398, 667)	656 (456, 919)	0.006^**^	559 (400, 778)	1,187 (567, 1,534)	0.022^*^
Lymphocyte count (×10^3^/μL)	1.3 (0.9, 1.9)	1.4 (0.9, 2.1)	1.3 (0.95, 1.8)	0.34	1.4 (0.97, 1.9)	1.00 (0.4, 2.02)	0.13
Lymphocytes (%)	18.9 (12, 28.0)	20.5 (13.2, 31.7)	16.9 (11.3, 25.3)	0.01^*^	18.9 (12.0, 28.1)	18.7 (12.1, 22.4)	0.5
Mean corpuscular hemoglobin concentration (g/dL)	34.2 (32.4, 35.3)	34.6 (32.7, 35.6)	33.9 (32.3, 35.0)	0.07	34.2 (32.5, 35.3)	33.5 (32.05, 35.6)	0.63
Mean cell hemoglobin (pg) (mean ± SD)	30.11 ± 3.44	30.18 ± 3.03	30.04 ± 3.77	0.73	30.06 ± 3.35	30.79 ± 4.67	0.38
Mean corpuscular volume (fL)	89.9 (85.6, 93.5)	89.3 (85.6, 92.9)	90.7 (85.8, 94.6)	0.11	89.9 (85.4, 93.4)	91.0 (87.8, 97.5)	0.11
Mean platelet volume (fL)	9.2 (8.6, 10.1)	9.3 (8.7, 10.1)	9.1 (8.5, 10.1)	0.40	9.2 (8.6, 10.0)	9.3 (8.67, 10.9)	0.34
Mixed cell count (×10^3^/μL)	0.4 (0.12, 0.7)	0.4 (0.2, 0.7)	0.4 (0.1, 0.6)	0.53	0.4 (0.19, 0.7)	0.2 (0, 0.65)	0.32
Mixed cell (%)	5.4 (3.0, 8.5)	6.0 (3.3, 9.2)	5.0 (3.0, 8.0)	0.07	5.4 (3.1, 8.6)	4.7 (2.2, 8.2)	0.13
Neutrophil count (×10^3^/μL)	4.5 (2.6, 7.6)	4.1 (3.0, 6.1)	4.7 (2.4, 8.3)	0.12	4.5 (2.6, 7.4)	5.2 (3.1, 8.7)	0.39
Neutrophil (%)	73.7 (62.0, 83.0)	71.3 (59.1, 80.9)	76.05 (63.6, 83.7)	0.02^*^	73.2 (61.6, 83.1)	76.1 (71.4, 81.6)	0.39
Potassium (mmol/L)	4.0 (3.8, 4.3)	4.0 (3.8, 4.3)	4.0 (3.7, 4.3)	0.80	4.0 (3.8, 4.3)	3.9 (3.4, 4.0)	0.29
P-LCR	21.6 (16.5, 27.8)	71.3 (59.1, 80.9)	21.3 (16.2, 27.5)	0.53	21.6 (16.5, 27.7)	22.2 (18.2, 33.2)	0.23
Platelet distribution width (%)	11.2 (9.9, 12.9)	11.3 (9.9, 12.9)	11.1 (9.8, 12.85)	0.74	11.2 (9.9, 12.8)	11.5 (9.9, 13.3)	0.71
Phosphorus (mg/dL)	3.3 (2.6, 4.1)	3.3 (2.8, 4.3)	3.3 (2.5, 4.0)	0.25	3.4 (2.7, 4.2)	2.7 (2.1, 3.2)	0.002^**^
Platelet count (×10^2^/μL)	1,820 (1,405, 2,435)	1,900 (1,480, 2,515)	1,790 (1,350, 2,410)	0.21	1,845 (1,410, 2,417)	1,490 (1,360, 4,875)	0.90
RBC count (×10^6^/μL) (mean ± SD)	4.39 ± 0.84	4.27 ± 0.82	4.49 ± 0.85	0.029^*^	4.41 ± 0.81	4.00 ± 1.15	0.047^*^
RDW – CV (%)	13.7 (13.1, 15.0)	13.6 (12.9, 14.4)	13.9 (13.3, 15.4)	0.007^**^	13.7 (13.2, 14.9)	13.9 (13.0, 15.9)	0.64
RDW – SD (fL)	47.3 (44.2, 50.6)	46.4 (43.3, 49.3)	48.3 (45.1, 52.3)	<0.001^*^	46.9 (44.0, 50.3)	49.2 (46.4, 51.9)	0.07
AST (U/L)	26 (18, 50)	24 (18, 47)	28 (19, 57)	0.23	26 (18, 47)	41 (20, 147)	0.12
Alanine transaminase (U/L)	25 (12, 47)	24 (13, 44)	26 (12, 52)	0.27	24 (12, 46)	29 (20, 69)	0.28
Sodium (mEq/L)	137 (135, 140)	138 (136, 140)	136 (134, 139)	0.008^**^	137 (135, 140)	135 (133, 138)	0.05
White blood cell count (/μL)	7,200 (5,400, 10,525)	7,050 (5,225, 9,650)	7,300 (5,400,10,675)	0.28	7,200 (5,400, 10,500)	7,600 (4,375, 11,475)	0.90

* *P* < 0.05;

** *P* < 0.01;

*** *P* < 0.001.

ALK-p, alkaline phosphatase; AST, aspartate aminotransferase; CRP, C-reactive protein; ESR, erythrocyte sedimentation rate; IQR, interquartile range; LDH, lactate dehydrogenase; P-LCR, platelet larger cell ratio; RBC, red blood cell; RDW, RBC distribution width; RDW-CV, RBC distribution width – coefficient of variation; RDW-SD, RBC distribution width – standard deviation; SARS-CoV-2, severe acute respiratory syndrome-coronavirus-2.

The results of univariate logistic regression analysis showed that older age, having an underlying disease, elevated LDH, hypophosphatemia, and change in RBC cell count were associated with both disease severity and mortality. Hypocalcemia was only associated with mortality (**Table 3**).

**Table 3:** Univariate logistic analysis of the severity and mortality of COVID-19

**Variable**	**Severity**			**Mortality**		
	
	**OR**	**Lower–upper**	***P***	**OR**	**Lower–upper**	***P***
Age	1.03	1.02–1.04	<0.001^***^	1.04	1.01–1.07	0.003^**^
Sex	1.02	0.63–1.65	0.942	0.79	0.31–2.06	0.63
Comorbidity	2.47	1.51–4.03	<0.001^***^	4.25	1.36–13.26	0.013^*^
Time from illness onset to first admission [median (IQR)]	1.07	1.00–1.15	0.48	1.12	1.02–1.23	0.013^*^
Time from admission to discharge/death [median (IQR)]	1.06	0.99–1.14	0.06	1.13	1.02–1.24	0.012^*^
Albumin	1.04	0.66–1.64	0.86	0.69	0.28–1.70	0.42
ALK-p	0.998	0.996–1.000	0.053	0.998	0.99–1.00	0.38
Direct bilirubin	0.92	0.57–1.50	0.74	1.11	0.55–2.27	0.77
Total bilirubin	1.08	0.77–1.52	0.66	1.20	0.82–1.78	0.35
Calcium	1.06	0.71–1.59	0.77	1.24	0.11–0.51	<0.001^***^
Corrected calcium	0.97	0.63–1.49	0.89	1.42	0.21–0.81	0.01^*^
CRP	1.01	0.98–1.04	0.58	0.98	0.88–1.09	0.64
ESR	1.01	0.996–1.016	0.27	1.00	0.98–1.02	0.80
Hematocrit	1.06	1.02–1.10	0.004^**^	1.03	0.96–1.12	0.40
Hemoglobin	1.12	1.01–1.24	0.030^*^	1.09	0.89–1.33	0.40
LDH	1.00	1.000–1.003	0.016^*^	1.00	1.000–1.001	0.024^*^
Lymphocyte count (/μL)	0.81	0.64–1.03	0.090	1.08	0.74–1.57	0.69
Lymphocyte percentage	0.97	0.95–0.99	0.004^**^	0.99	0.94–1.03	0.50
Mean corpuscular Hb concentration	0.95	0.86–1.05	0.30	0.95	0.79–1.14	0.55
Mean cell hemoglobin	0.99	0.92–1.06	0.73	1.07	0.92–1.25	0.38
Mean cell volume	1.01	0.98–1.04	0.53	1.05	0.98–1.12	0.19
Mean platelet volume	0.97	0.91–1.04	0.39	1.01	0.94–1.08	0.76
Mixed cell count	0.84	0.58–1.23	0.38	0.79	0.24–2.55	0.69
Mixed cell percentage	0.95	0.90–1.00	0.062	0.89	0.77–1.03	0.12
Neutrophil count	1.06	1.01–1.13	0.03^*^	1.04	0.95–1.13	0.42
Neutrophil percentage	1.01	0.996–1.021	0.17	1.01	0.98–1.03	0.61
P-LCR	0.996	0.97–1.02	0.75	1.04	0.99–1.10	0.11
Platelet distribution width	1.00	0.93–1.07	>0.99	1.12	0.95–1.32	0.18
Phosphorus	0.82	0.66–1.01	0.063	0.36	0.19–0.69	0.002^**^
Platelet count (×10^3^/μL)	1	1–1	0.96	1.00	1.000–1.000	0.010^*^
Potassium	0.97	0.57–1.65	0.91	0.30	0.09–0.97	0.045^*^
RBC count	1.39	1.03–1.88	0.033^*^	0.62	0.39–1.00	0.049^*^
RDW–CV	1.20	1.06–1.37	0.005^**^	1.04	0.84–1.29	0.71
RDW–SD	1.06	1.01–1.10	0.009^**^	1.03	0.97–1.10	0.34
AST	1.01	1.00–1.01	0.074	1.00	1.00–1.01	0.14
Alanine transaminase	1.01	0.999–1.011	0.14	1.00	0.998–1.003	0.81
Sodium	0.94	0.891–0.998	0.043^*^	0.90	0.80–1.01	0.07
White blood cell count (/μL)	1.00	1.00–1.00	0.59	1.00	1.00–1.00	0.89

* *P* < 0.05;

** *P* < 0.01;

*** *P* < 0.001.

ALK-p, alkaline phosphatase; AST, aspartate aminotransferase; COVID-19, coronavirus disease-2019; CRP, C-reactive protein; ESR, erythrocyte sedimentation rate; IQR, interquartile range; LDH, lactate dehydrogenase; OR, odds ratio; P-LCR, platelet larger cell ratio; RBC, red blood cell; RDW-CV, RBC distribution width – coefficient of variation; RDW-SD, RBC distribution width – standard deviation.

Multivariate logistic regression found that older age [odds ratio (OR) 1.02, 95% CI 1.01–1.04, *P* = 0.001], presence of comorbidities (OR 1.86, 95% CI 1.03–3.36, *P* = 0.04), increase in hematocrit (OR 1.08, 95% CI 1.03–1.13, *P* = 0.001), and increase in RBC distribution width – coefficient of variation (RDW-CV) (OR 1.18, 95% CI 1.02–1.36, *P* = 0.02), were significantly associated with disease severity (**Table 4**). Older age (OR 1.00, 95% CI 1.00–1.07, *P* = 0.03), hypocalcemia (OR 0.20, 95% CI 0.09–0.58, *P* = 0.002), hypophosphatemia (OR 0.50, 95% CI 0.26–1.02, *P* = 0.04), and increase in platelet larger cell ratio (P-LCR) (OR 1.10, 95% CI 1.00–1.15, *P* = 0.04) were significantly associated with mortality (**Table 5**).

**Table 4:** Logistic regression of risk factors related to severity of COVID-19

**Variable**	**B**	**SE**	**OR**	**95% CI**	**Wald statistic**	***P***
Age	0.23	0.007	1.02	1.01–1.04	10.5	0.001^**^
Hematocrit	0.076	0.025	1.08	1.03–1.13	9.42	0.002^**^
RDW-CV	0.162	0.073	1.18	1.02–1.36	4.95	0.02^*^
Comorbidity	0.619	0.303	1.86	1.03–3.36	4.19	0.04^*^

* *P* < 0.05;

** *P* < 0.01;

*** *P* < 0.001.

B, logistic regression coefficient (β); CI, confidence interval; COVID-19, coronavirus disease-2019; OR, odds ratio; RBC, red blood cell; RDW-CV, RBC distribution width – coefficient of variation; SE, standard error for the unstandardized β.

**Table 5:** Logistic regression of risk factors related to mortality of patients with COVID-19

**Variable**	**B**	**SE**	**OR**	**95% CI**	**Wald statistic**	***P***
Calcium	−1.453	0.466	0.20	0.09–0.58	9.726	0.002^**^
Phosphorus	−0.662	0.346	0.50	0.26–1.02	3.651	0.04^*^
P-LCR	0.069	0.035	1.10	1.00–1.15	994	0.04^*^
Age	0.034	0.017	1.00	1.00–1.07	4.039	0.03^*^

* *P* < 0.05;

** *P* < 0.01;

*** *P* < 0.001.

B, logistic regression coefficient (β); CI, confidence interval; COVID-19, coronavirus disease-2019; OR, odds ratio; P-LCR, platelet larger cell ratio; RBC, red blood cell; SE, standard error for the unstandardized β.

**Table 6:** Area under the ROC curve and optimal threshold of each COVID-19-related variable

**Variables**	**AUC**	**95% CI**	***P***	**Optimal threshold**	**Sensitivity**	**Specificity**
Calcium	0.759	0.69–0.82	0.001^**^	≤6.4	64.7	59.1
LDH	0.731	0.64–0.81	0.039^*^	>4,622	55.6	92.7
Phosphorus	0.725	0.66–0.79	<0.001^**^	≤1.5	76.5	69.2
Bilirubin Total	0.689	0.61–0.76	0.003^**^	>4.8	66.7	69.4
Total CRP	0.679	0.62–0.74	0.013^*^	>90	53.3	83.1
RDW SD	0.624	0.56–0.68	0.035^*^	>79.3	88.9	38.2

* *P* < 0.05;

** *P* < 0.01;

*** *P* < 0.001.

AUC, area under the curve; CI, confidence interval; COVID-19, coronavirus disease-2019; CRP, C-reactive protein; LDH, lactate dehydrogenase; RDW-SD, red blood cell distribution width – standard deviation; ROC, receiver operating characteristic.

Spearman rank correlation analysis revealed the following: a positive correlation between the serum levels of calcium and phosphorus on admission and the prognosis of patients with COVID-19 (*r* = 0.25 and 0.21, respectively; *P* < 0.05); and a negative correlation between the serum levels of LDH, bilirubin direct, and bilirubin total values and the prognosis of patients with COVID-19 (*r* = − 0.22, −0.2, and −0.21, respectively; *P* < 0.05).

The predictive value of laboratory test results for hospital mortality were calculated (**Table 3**).

Abnormalities on chest radiography (CR) and/or CT were detected in all patients. Bilateral involvement was noted in 245 (85.7%) of the chest radiographs or CTs.

Typical chest CT images of infected patients demonstrate multiple, bilateral, patchy ground-glass opacities with peripheral and subpleural distribution.

All patients received supportive therapy, antiviral treatment including hydroxychloroquine sulfate 400 mg/d orally for 7–10 d or and chloroquine phosphate (500 mg twice daily, orally) along with Kaletra (lopinavir/ritonavir) two 200 mg tablets twice daily, for 5–14 d. Antibiotics and methylprednisolone were used for some patients.

Of the 286 enrolled patients with COVID-19, 45 (15.7%) patients had secondary infection with bacterial and fungal infections; further, among this group of patients with secondary infections, nonsurviving patients had higher rates of secondary bacterial infections than patients who survived (10.3% vs. 5.2%, *P* = 0.007). However, clinicians did report that secondary bacterial infection could be as high as 50% among nonsurvivors.

## Discussion

In this retrospective cohort study, we identified the risk factors for mortality among 286 patients with COVID-19 who were admitted to the Hajar Hospital affiliated with the Shahrekord University of Medical Sciences. Older age, underlying comorbidities, elevated hematocrit, and increased RDW-CV levels on admission were associated with severe disease, while older age, hypocalcemia, hypophosphatemia, and elevated P-LCR levels were associated with an increased risk of death in the hospital. Adapted to a hypoxic environment, patients at high altitudes such as in Shahr-e Kord might react differently from people at lower altitudes, and treatment for patients in high-altitude regions may need special consideration [6].

Some studies showed that male patients with COVID-19 have a higher risk of severe disease and death compared with female patients, but our results show that sex may not be a risk factor for the mortality following COVID-19 infection; this observation is consistent with other reports [15], and it seems that the finding is a result of the small sample size.

Older age was associated with poor condition and outcome in patients with COVID-19. The age of deceased patients was greater than that of survivors. Previous studies have found that older adults and people with underlying medical conditions have a worse prognosis with COVID-19 [16]. Reduced humoral immunity and the age-related loss of the numbers and function of naïve T cells is an explanation for the mortality associated with infection and long-lasting inflammation.

The risk of COVID-19 is higher in patients who have a comorbid condition, such as hypertension, diabetes mellitus, heart disease, and cancer. Patients with chronic conditions might also be more likely than others to become severely ill and die [17, 18].

The present study shows that hypertension, coronary artery disease, chronic kidney disease, and chronic obstructive pulmonary disease (COPD) are the most prevalent underlying or comorbid conditions among patients with COVID-19 who did not survive.

The main characteristics of patients with COVID-19, as determined by laboratory test results on admission, were as follows: normal ranges of white blood cell count, neutrophil count, and PLT count; decreased serum level of calcium; and increased serum levels of CRP, ALK-p, direct bilirubin, LDH, AST, and ALT, and increased red blood cell (RBC) distribution width – standard deviation (RDW-SD) and ESR. We found that hypoalbuminemia and anemia occurred in the course of disease in many patients. Hypoalbuminemia in severe COVID-19 has been addressed repeatedly in the literature [19,20,21,22,23].

We found an association between laboratory test results on admission and COVID-19 severity level and poor outcomes. The serum levels of CRP and LDH were significantly higher in deceased patients than in patients who survived, but the serum levels of calcium and phosphorus in deceased patients were lower than in patients who survived. Lymphopenia has been suggested to predict the severity of COVID-19 [24]. However, we found no significant correlation between lymphopenia and the prognosis of patients with COVID-19. In the present study, ROC curves were drawn to determine the prognostic value of laboratory indicators. The AUC of the ROC curves for RDW-SD, CRP, and total bilirubin ranged from 0.62 to 0.68. The highest AUC was for serum calcium level. The optimal working point was 6.4 mg/L, with 64.7% sensitivity and 54.2% specificity for predicting the prognosis of patients with COVID-19. The AUC for LDH was 0.73. The optimal working point was 4,622 mg/L, with 55% sensitivity and 92% specificity for predicting the prognosis of patients with COVID-19. CRP is a useful inflammatory marker and indicator that plays an essential role in the host's resistance against pathogens and inflammation [25]. We used the laboratory data on admission; so, the patient might have had an elevated CRP level during the hospital admission and then be mechanically ventilated. CRP levels are positively correlated with the size of lung injury and severe presentation in SARS-CoV-2-infected patients [26]. Higher CRP levels are associated with unfavorable features of COVID-19 diseases, such as respiratory failure, acute heart injury, and fatality [27,28,29].

Associations between elevated RDW and risk of mortality in patients hospitalized with COVID-19 have been reported [30]. The mechanism underlying the role of RDW in COVID-19 disease remains unclear. RDW is a nonspecific and general biomarker of disease [31,32,33,34,35,36,37,38,39,40] and is therefore unlikely to be causally related to the progression of COVID-19-related pneumonia.

Turnover of the various types of leukocyte lineages is altered in patients with COVID-19, and changes in the number and dynamics of PLTs in coagulopathy are associated with COVID-19 [41]. We found an association of elevated RDW with the severity of COVID-19 disease, which is consistent with previous studies (in non-COVID-19 cohorts), which in turn suggested that the RDW levels increase when the kinetics of RBC production have slowed in the setting of increased PLT or leukocyte production or increased PLT or leukocyte turnover, such as would occur in inflammation [42, 43].

Hypocalcemia has been commonly observed among patients hospitalized with COVID-19 and carries a poor prognosis [11, 44,45,46]. The cause of hypocalcemia in patients with COVID-19 is not clear, but is likely to be multifactorial, including calcium-dependent viral mechanisms of action, hypoproteinemia, imbalanced vitamin D and parathyroid hormone levels in the acute phase of COVID-19 infection, chronic and acute malnutrition during critical illness, and high levels of unbound and unsaturated fatty acids in inflammatory responses. The underlying disease of the patients who did not survive was not apparently associated with low serum levels of calcium (*P* = 0.62) or low phosphorus (*P* = 0.70).

Numerous studies have shown a strong correlation between hypocalcemia and inflammatory markers that reflect COVID-19 severity. Thus, as in tumor lysis syndrome, serum calcium levels were negatively correlated with plasma levels of CRP [44, 45], LDH [45], interleukin-6, and procalcitonin [46], which support viral replication, development of a cytopathic effect, and subsequent cell death [47]. The time-to-hospitalization and time-from-admission-to-discharge/death were associated with clinical outcomes (**Table 1**).

The present study has several limitations. First, the study was conducted with patient data from a single center in a high-altitude region of Iran, and some patients suspected with COVID-19 had a clinical rather than RT-PCR assay diagnosis, which may affect the generalizability of the study results. Second, the sample size was small, especially in the subgroup of patients who did not survive. Third, the CRP level had not been monitored at hospital admission, and the effect of medication, including corticosteroids and immunomodulators, on clinical outcomes was not evaluated. At the time that the patients were treated, effective drugs, vaccines, or standard therapeutic procedures were not available. Empirical therapy was being used to manage and save the life of patients with known antivirals, antibiotics, and corticosteroids, either alone or in combination, based on the patient's condition, need, and availability, along with other supportive therapies [48, 49]. Several medications used for the treatment of COVID-19 have uncertain safety and efficacy profiles. Hydroxychloroquine, chloroquine, and lopinavir/ritonavir have quite limited effectiveness, and the potential cardiovascular effects of these drugs may have affected the outcomes, particularly in patients with cardiac comorbidity [50].

## Conclusions

Predictors of a fatal outcome for patients with COVID-19-related pneumonia at admission include serum calcium and phosphorus levels and P-LCR. Among these factors, calcium level was the strongest single laboratory predictor of mortality in patients with COVID-19. Patients at high altitudes such as in Shahr-e Kord might respond to the disease differently from people at lower altitudes, and treatment for patients in high-altitude regions may need special consideration.
